# The effects of alcohol and marijuana on survival after severe traumatic brain injury: A retrospective cohort study

**DOI:** 10.1016/j.amsu.2020.07.031

**Published:** 2020-07-22

**Authors:** John J. Leskovan, Puja D. Patel, John M. Pederson, Aaron Moore, Amer Afaneh, Laura R. Brown

**Affiliations:** a Department of Trauma Surgery, Mercy St. Vincent Medical Center, Toledo, OH, USA; b Superior Medical Experts, Minneapolis, MN, USA; c Department of Surgery, MetroHealth Medical Center, Cleveland, OH, USA

**Keywords:** Abbreviated injury scale, Alcohol consumption, Logistic models, Marijuana, Tetrahydrocannabinol, Traumatic brain injury

## Abstract

**Background:**

Alcohol (ETOH) and marijuana (THC) use have previously shown to improve outcomes after Traumatic Brain Injury (TBI). However, whether TBI severity impacts outcomes among patients tested positive for both ETOH and THC remains unclear.

**Materials and methods:**

A retrospective review from the Northern Ohio Regional Trauma Registry, which includes deidentified data from six regional hospitals, including three Level 1 and three Level 3 trauma centers, was performed to assess adult (>18 years) patients with severe TBI (head Abbreviated Injury Score ≥ 3) between January 2012 and December 2018 having an alcohol and drug toxicology screen and data regarding outcome at discharge. Patients were divided into two groups: 1) patients with a negative ETOH and drug test, and 2) patients positive for ETOH + THC. Mortality at discharge was the primary outcome measure and multiple logistic regression was used to assess predictors of mortality at discharge.

**Results:**

A total of 854 (median age: 51 years [range: 18–72]; 34.4% female [294/854]) patients were included. On multiple logistic regression, age (p = 0.003), days in intensive care unit (ICU) (p < 0.001), Glasgow Coma Scale (GCS) (p < 0.001), Injury Severity Score (ISS) (p < 0.001), length of stay (LOS) (p < 0.001), and days on ventilator support (p = 0.032) were significant predictors of mortality at discharge. Blood alcohol content (BAC), cause of TBI, drug class, and sex were not significant predictors of mortality at discharge.

**Conclusions:**

After severe TBI, positive THC and BAC screening did not predict mortality at discharge after controlling for confounding variables, indicating no survival benefit for patients with severe TBI.

## Highlights

• No survival benefit of alcohol and marijuana for severe traumatic brain injury.
• Glasgow Coma Scale predict mortality for severe traumatic brain injury.
• Injury Severity Score predict mortality for severe traumatic brain injury.

## 1 Introduction

Traumatic brain injury (TBI) is associated with a mortality rate of up to 35.8%, and alcohol (ETOH) intoxication has been shown to be present in 30–50% of TBIs [[1], [2], [3]]. Recently, marijuana (tetrahydrocannabinol or THC) was shown to be associated with decreased mortality rates for adult patients after TBI: A 3-year retrospective review of data from a Level 1 trauma center found that positive THC screening was independently associated with survival after TBI. A head Abbreviated Injury Scale (AIS) ≥ 5 has been shown to be an independent predictor of poor outcome after TBI and both alcohol and THC have shown neuroprotective effects for TBI [4,5]. The aim of this study is to examine the effects of positive THC toxicology and blood alcohol content (BAC) screens on outcomes after severe TBI by utilizing a multi-center dataset from 26 regional hospitals.

## 2 Materials and methods

This work has been reported in accordance with the STROCSS criteria [6].

### 2.1 Study population

Institutional Review Board approval was obtained to analyze deidentified patient data from the Northern Ohio Regional Trauma Registry collected between January 1, 2012 to December 31, 2018. Data was retrospectively reviewed and screened for patients who met the inclusion criteria of TBI with Head AIS ≥3, age ≥18 years, a toxicology screen, drug screen results, and documented data regarding outcome at discharge. Exclusion criteria included pediatric patients (age <18 years) and patients without toxicology screen or results and discharge outcomes. Patients were excluded if they tested positive for drugs other than ETOH and THC, including amphetamines, barbiturates, benzodiazepines, cocaine, methamphetamine, 3,4-methylenedioxymethamphetamine (MDMA), methadone, opioids, oxycodone, phencyclidine, and tricyclic anti-depressants.

Included patients were divided into two groups: 1) No Substances (negative toxicology and BAC test), and 2) ETOH + THC (positive toxicology for THC and blood alcohol and negative for all other drugs).

### 2.2 Study variables

Patient data collected included age, gender, Glasgow Coma Scale (GCS), Head AIS, Injury Severity Score (ISS), complications, and mechanism of injury. Outcome variables included days on ventilator support, days in intensive care unit (ICU), length of hospital stay (LOS, days), mortality, and discharge disposition.

### 2.3 Statistical analyses

Fisher's exact test was used for comparisons of dichotomous data [7]. Odds ratios and 95% CIs were also computed using the Woolf logit method. The Mann-Whitney *U* test was used to compare continuous and ordinal-scale data [8]. Multiple logistic regression was used to identify predictors of discharge mortality rates. Multiple imputation using chained equations through predictive mean matching was used to handle missing data in the regression model. P-values from logistic regression are computed via Wald's test [9]. P-values ≤0.05 were considered statistically significant. Statistics were performed in RStudio (Version February 1, 5033).

## 3 Results

A total of 854 patients met our inclusion criteria and were included in the analysis, with 166 (19.4%) in the ETOH + THC group and 688 (80.4%) in the No Substances group (Table 1). Comparisons of sex and mortality at discharge by group were performed (Table 2). Significant differences for sex were found between the ETOH + THC and No Substances groups (OR 0.424 [95% CI: 0.2829 to 0.6313], p < 0.001) with more females in the No Substances group. There were no significant differences between groups for mortality at discharge.

**Table 1:** Patient characteristics by group.

Characteristic	ETOH + THC (N = 166)	No Substances (N = 688)
Age, median (IQR)	34 (18–48)	56 (35–72)
Female, n (%)	34 (20.48%)	260 (37.79%)
Complications, n	37	119
GCS, median (IQR)	14 (7–15)	15 (13–15)
ISS, median (IQR)	9 (5–17)	10 (5–20)
ICU, median (IQR)	1 (0–3)	1 (0–3)
LOS, median (IQR)	2 (1–5)	3 (1–6)
Ventilator Days, median (IQR)	0 (0–1)	0 (0–1)

Data are mean ± SD, n (%), or median (IQR). EtOH = Alcohol; THC = tetrahydrocannabinol; GCS = Glasgow Coma Scale; ICU=Intensive Care Unit; ISS=Injury Severity Score; LOS = Length of stay.

**Table 2:** Dichotomous comparisons of sex and mortality at discharge by group.

	Female	Male	Total	Odds Ratio	95% CI	P-value
Sex						
ETOH + THC	34	132	166	0.424	0.2829–0.6313	<0.001
No Substances	260	428	688			
**Mortality at Discharge**						
ETOH + THC	9	157	166	0.569	0.2826–1.1440	0.160
No Substances	63	625	688			

THC = tetrahydrocannabinol; CI=Confidence Interval.

Comparisons of background characteristics included age, LOS (days), ICU stay (days), ventilator (days), GCS, and ISS (Table 3). Significant differences in age were found between the ETOH + THC and No Substances groups (p < 0.001) with an older median age in No Substances group. Patients in the No Substances groups had a significantly longer median LOS and greater median GCS scores than those in the ETOH + THC group (p < 0.001 and p < 0.001, respectively).

**Table 3:** Comparisons of background characteristics (ETOH + THC vs. No Substances).

	ETOH + THC (n = 166)	No Substance (n = 688)	Diff. Actual	Diff. Hodges-Lehmann	U	p-value
Age	34	56	22	18	32251	<0.001
LOS (days)	2	3	1	1	47726	<0.001
ICU (days)	1	1	0	0	54997	0.443
Ventilator (days)	0	0	0	0	52852	0.082
GCS	14	15	1	0	46779	<0.001
ISS	9	10	1	0	54378	0.337

Data are reported as medians. ETOH = Alcohol; THC = tetrahydrocannabinol; LOS = length of stay; ICU=Intensive care unit; GCS = Glasgow Coma Scale; ISS=Injury Severity Score.

### 3.1 Multiple logistic regression

On multiple logistic regression the following variables were identified as significant predictors of mortality at discharge: Age (OR = 1.043 [95% CI: 1.016–1.074], p = 0.003), ICU days (OR = 1.636 [95% CI: 1.261–2.145], p < 0.001), GCS (OR = 0.755 [95% CI: 0.684–0.827], p < 0.001), ISS (OR = 1.136 [95% CI: 1.091–1.189], p < 0.001), LOS days (OR = 0.427 [95% CI: 0.321–0.543], p < 0.001), and ventilator days (OR = 1.310 [95% CI: 1.044–1.711], p = 0.032). BAC, cause of TBI, drug class (ETOH + THC, no substances), and sex were not significant predictors of mortality at discharge. McFadden's Pseudo R^2^ value of the multiple logistic regression model was 0.655 (p < 0.001), implying mortality at discharge can be reliably predicted by the model (Table 4).

**Table 4:** Logistic regression.

	Log Odds Ratio	Standard Error	Z value	p-value
Intercept (log odds)	−2.307	1.821	−1.267	0.205
Age	0.042	0.014	3.005	0.003
Sex	0.104	0.472	0.220	0.826
BAC	−3.604	5.090	−0.708	0.479
ICU (days)	0.492	0.136	3.629	<0.001
Group: No Substances	−1.110	1.188	−0.934	0.350
GCS	−0.281	0.048	−5.832	<0.001
ISS	0.127	0.022	5.838	<0.001
LOS (days)	−0.850	0.133	−6.397	<0.001
Ventilator (days)	0.270	0.126	2.150	0.032
**Cause**				
Fall	0.116	1.313	0.088	0.930
Firearm	1.026	1.824	0.562	0.574
MCC	−0.705	1.412	−0.499	0.617
MVC	−0.477	1.330	−0.359	0.720
Other	1.114	1.491	0.748	0.455
Pedestrian	1.205	1.636	0.736	0.461
Struck/Assault	−1.542	1.739	−0.886	0.376
	**Null Deviance**	**Residual Deviance**	**McFadden's Pseudo R**^**2**^	**p-value**
**Summary**	493.90 (df = 853)	170.61 (df = 837)	0.655	<0.001

BAC=Blood alcohol content; MCC = Motorcycle crash; MVC = Motor vehicle collision; GCS = Glasgow Coma Scale; ICU=Intensive Care Unit; ISS=Injury Severity Score; LOS = Length of stay; THC = tetrahydrocannabinol.

## 4 Discussion

Our results demonstrate patients with severe TBI with positive ETOH and THC have no difference in mortality at discharge compared to patients with no substances. On multiple logistic regression, age, ICU days, GCS, ISS, LOS days, and ventilator days were found to be independent predictors of mortality, while BAC, cause of TBI, drug class, and sex were not independent predictors of mortality. In addition, despite injuries being worse according to GCS scores in the ETOH + THC compared to the No Substances group, the No Substances group had a longer median LOS; this finding may be attributed to the older age distribution in the No Substances group, as older patients are known to have longer recovery times after TBI [10]. GCS scores measured in intoxicated patients, including positive BAC, THC, benzodiazepines, opiates, and cocaine, have also been shown to be confounded, possibly affecting performance metrics and predictive analytics [11].

Higher head AIS scores have been associated with higher rates of mortality and functional disability after TBI [5,12]. Some studies demonstrated that alcohol intoxication in patients with severe TBI may improve mortality rates: A meta-analysis by Raj et al. including 95,941 patients found that positive BAC was significantly associated with lower mortality rates in moderate to severe TBI [13]. Another study of 352 patients with severe TBI by Mohseni et al. found that positive BAC on admission was associated with better long term functional outcome [14]. In our study, a positive BAC was not associated with decreased mortality rates.

Increased use of marijuana among Americans is coincided with its implication in emergency departments visits, motor vehicle collisions, and trauma [15,16]. THC has been associated with neuroprotective effects after TBI in several preclinical studies [17,18]. A 3-year retrospective review from a Level 1 trauma center by Nguyen et al. found that positive THC screens were associated with improved survival after TBI [19]. Ages ≥45 years and AIS >4 were also found to be independent predictors of mortality, though the effects of THC on patients with head AIS >4 was not explicitly stated by Nguyen et al. Our study is the first to consider both positive THC and BAC screening on admission in patients with severe TBI and find that their combination does not predict mortality at discharge. Differences in outcomes between our study and that of Nguyen et al. is partially due to differences in study populations and variables accounted for in our statistical models. Nguyen et al. employed separate logistic regressions and sorted data by dichotomizing background characteristics, while our study used multiple logistic regression, including undichotomized data, including the full range of possible values. Unnecessary dichotomization can have considerable consequences, and various studies on regression model methodologies firmly favor use of undichotomized and unreduced data [20].

Our study has several limitations, including limited data on complications during hospital stay and absence of data on treatments used and causes of death. Additionally, we were not able to distinguish between chronic and acute drug use because past drug history was not collected. Limitations in toxicology screens may yield positive THC screening for patients who recently used THC, or 4.6–15.4 days previously [21]. Our end point was mortality at discharge, which did not consider the long-term effects of BAC after TBI. Furthermore, our analysis did not evaluate effects of THC and ETOH on mortality in TBI patients independently; thus, our results can only be applied to patients tested positive or negative for both THC and ETOH.

## 5 Conclusions

We found that after severe TBI, positive BAC and THC screening was not an independent predictor of mortality at discharge after controlling for confounding variables among patients from a multi-center registry from Level 1 and Level 3 trauma centers.

## Funding

This research did not receive any specific grant from funding agencies in the public, commercial, or not-for-profit sectors.

## Ethical approval

Institutional Review Board approval was obtained to analyze deidentified patient data.

## Consent

This manuscript does not contain patient-level data or any identifiable patient information. Institutional Review Board approval was obtained to analyze deidentified patient data.

## Registration of research studies

1. Name of the registry: Research Registry.

2. Unique Identifying number or registration ID: researchregistry5555.

3. Hyperlink to your specific registration (must be publicly accessible and will be checked): https://www.researchregistry.com/browse-the-registry#home/registrationdetails/5eaad9846f443f00162e334f/

## Guarantor

John J. Leskovan, DO.

Department of Trauma Surgery.

Mercy St. Vincent Medical Center.

2213 Cherry St, Toledo OH, 43608.

Email: leskovjj@gmail.com.

Telephone: 419-251-4674.

Fax: 419-251-3862.

## Provenance and peer review

Not commissioned, externally peer reviewed.

## CRediT authorship contribution statement

**John J. Leskovan:** Conceptualization, Supervision, Methodology, Writing - review & editing. **Puja D. Patel:** Writing - original draft, Writing - review & editing. **John M. Pederson:** Methodology, Investigation, Formal analysis, Data curation, Writing - review & editing, Visualization. **Aaron Moore:** Writing - review & editing. **Amer Afaneh:** Writing - review & editing. **Laura R. Brown:** Writing - review & editing.

## Declaration of competing interest

PDP and JP contract with Superior Medical Experts. The authors report no conflict of interest concerning the materials or methods used in this manuscript.
